# Detection of DNA Methyltransferase Activity via Fluorescence Resonance Energy Transfer and Exonuclease-Mediated Target Recycling

**DOI:** 10.3390/bios12060395

**Published:** 2022-06-08

**Authors:** Tingting Hu, Changbei Ma, Ying Yan, Junxiang Chen

**Affiliations:** 1 School of Life Sciences, Central South University, Changsha 410013, China; hutingting516@163.com (T.H.); macb2012@csu.edu.cn (C.M.); yany2018@csu.edu.cn (Y.Y.); 2 Department of Urology, The Second Xiangya Hospital of Central South University, Changsha 410011, China

**Keywords:** DNA methyltransferase, cationic conjugated polymers, fluorescence resonance energy transfer, exonuclease III-assisted signal amplification

## Abstract

In this study, a sensitive method for detecting DNA methyltransferase (MTase) activity was developed by combining the effective fluorescence resonance energy transfer (FRET) of cationic conjugated polymers and exonuclease (Exo) III–mediated signal amplification. DNA adenine MTase targets the GATC sequence within a substrate and converts the adenine in this sequence into N6-methyladenine. In the method developed in this study, the methylated substrate is cleaved using Dpn I, whereby a single-stranded oligodeoxynucleotide (oligo) is released. Afterward, the oligo is hybridized to the 3ʹ protruding end of the F-DNA probe to form a double-stranded DNA, which is then digested by Exo III. Subsequently, due to weak electrostatic interactions, only a weak FRET signal is observed. The introduction of the Exo-III–mediated target-recycling reaction improved the sensitivity for detecting MTase. This detection method was found to be sensitive for MTase detection, with the lowest detection limit of 0.045 U/mL, and was also suitable for MTase-inhibitor screening, whereby such inhibitors can be identified for disease treatment.

## 1. Introduction

As one of the most important epigenetic modifications, DNA methylation highly affects gene expression, genomic stability, cell growth, and cell senescence [1,2]. DNA methyltransferases (MTases) transfer the methyl group of S-adenosine methionine (SAM) to the N6 position of adenine, or C5 or N4 position of cytosine, thereby protecting the DNA substrate from catalysis by restriction enzymes [3]. Although the nucleotide sequence does not change after DNA methylation, the expression of the corresponding gene is affected. MTases are involved in numerous complex biological events, such as aging, Alzheimer’s disease, and tumorigenesis [4]. DNA-methylation tests show 95% accuracy in detecting cancers. In addition, such tests can identify 97% and 94% of liver and lung metastases from colorectal cancer, respectively [5]. As a potential biomarker, abnormal MTase activity leads to a wide range of physiological or pathological changes. Therefore, the development of a highly sensitive and specific method for the detection of MTase activity is of great significance and has increasingly attracted attention.

The traditional methods used for detecting MTase activity include gel electrophoresis, high-performance liquid chromatography, and radioisotope-labeling strategies [6,7]. Although these methods are well established, most of them either are labor-intensive or are involved the use of radioactive materials. Several new methods, such as a colorimetric method based on G-quadruplex, and one based on DNA-gold nanoparticles with electrochemical biosensors [8,9], have been developed to overcome these shortcomings. However, these strategies either have high detection limits or involve monotonous nanoparticle preparation [7]. Thus, fluorescence-based assays have been widely sought to develop a method with improved detection for MTase activity. 

As a cationic conjugated polymer, poly (9,9-bis(6′-N,N,N-trimethylammoniu m)hexyl)-fluorenylenephenylenedibromide (PFP), has attracted great attention due to its unique fluorescence properties [10,11,12]. This cationic polymer is positively charged and can adsorb to double-stranded DNA molecules through electrostatic interactions, thereby enhancing the solubility of the adsorbed DNA in aqueous solutions. Therefore, such polymers are typically used in the detection of molecules, such as biomolecules [12,13].

In this study, we developed a simple and specific strategy to detect MTase activity. HP-DNA containing a sequence of GATC was used as the methylation substrate of MTase. Once the HP-DNA was methylated by MTase, it could be recognized and cleaved by Dpn I, which allows the release of a single-stranded DNA. This exonuclease-assisted fluorescence assay is based on effective fluorescence resonance energy transfer (FRET). FRET occurs when the emission spectrum of the donor molecule overlaps with the excitation spectrum of the receptor molecule. Due to its high sensitivity and selectivity, FRET has been used for detecting various molecules [13,14,15,16]. In this study, a hairpin probe labeled with a donor fluorophore carboxyfluorescein (FAM) at the 3′-terminal was employed to evaluated MTase activity. The FRET between PFP and FAM was used. For maximum detection sensitivity, nuclease-based DNA amplification techniques, such as rolling circle amplification, polymerase chain reaction, and DNA chain shift polymerization, have previously been used [17,18,19,20]. In this study, we used an exonuclease-assisted signal amplification strategy involving the sequence-independent enzyme Exonuclease III (Exo III). This enzyme sequentially excises the nucleotides of a duplex DNA with a blunt or recessed 3ʹ-terminus only from the 3′-terminus [17,21]. Even a small template amount could yield a high target signal. The method developed in this study takes advantage of an exonuclease-assisted fluorescence assay based on FRET and exhibits high sensitivity in detecting DNA MTase activity. Importantly, the amount and activity of MTase directly affect the level of DNA methylation, abnormal expression of DNA MTase has been found in a variety of tumor cells, and DNA MTase inhibitors have potential applications in antibiotics and anticancer therapy. Via a drug-screening approach based on this method, we identified inhibitors of MTase and then evaluated their effects in actual biological samples, thereby showing the potential of this method for biomedical research and clinical applications.

## 2. Materials and Methods

### 2.1. Materials and Reagents

DNA adenine methyltransferase (MTase), S-Adenosyl-L-methionine (SAM), Uracil-DNA glycosylase (UDG), Dpn I, Nb.BtsI and Exonuclease III were all bought from from Takara Biotech-nology Co., Ltd. (Dalian, China). poly (9,9-bis(6′-N,N,N-trimethylammonium)hexyl)-fluorenylenephenylenedibromide (PFP) and 5-fluorouracil were bought from Yuanye Co., Ltd. (Shanghai, China). Two oligonucleotides were synthesized by Shanghai Sangon Biotech Co., Ltd. (Shanghai, China). Carboxyfluorescein-labeled DNA (F-DNA): 5′-AGGAAGACGTACGTATCTTCC TCTAATGA-FAM-3′, HP-DNA: 5′-CCCAACGGATCATTAGAGGAAGATACGTACAA TGATCCGTTGGTATAT-3′.

### 2.2. Apparatus

All fluorescence intensity was measured using an F-2700 fluorescence spectrophotometer (Hitachi Ltd., Hitachi, Japan) at 25 °C. Both the excitation and emission slits were set at 10.0 nm, and the photomultiplier tube voltage was set at 700 V. The emission spectrum of 400–600 nm was collected following excitation at 380 nm. 

### 2.3. Optimization of the Experimental Conditions

To achieve the best performance, the optimal F-DNA, HP-DNA, Dpn I, and Exo III concentrations within 50–200 nM, 15–120 nM, 20–100 U/mL, and 0.1–8 U/mL, respectively, were determined.

### 2.4. Detection of MTase

To measure the activity of MTase quantitatively, the reaction buffer (10 mM Tris-HCl, 50 mM NaCl, 10 mM MgCl_2_, and 1 mM DTT; pH 7.9) containing 50 nM HP-DNA and various concentrations of MTase ranging from 0 to 30 U/mL were incubated at 37 °C for 60 min. Next, the mixture was incubated with 125 nM F-DNA at 37 °C for 60 min. Afterward, 2 U/mL Exo III was added, and the mixture was incubated at 37 °C for 30 min. Then, 1 μM PFP was added, and the mixture was incubated at room temperature for 20 min. Finally, the fluorescence spectra of all the samples were monitored by the F-2700 fluorescence spectrophotometer. To evaluate the practical application of this assay, we detected different concentrations of MTase in diluted serum.

### 2.5. Inhibition Assay of MTase Activity

For the MTase-activity inhibition assay, various amounts of 5-fluorouracil were added to the reaction before the addition of 40 U/mL MTase. After the reaction was performed as described above, the fluorescence intensity of 5-fluorouracil at each concentration was recorded at room temperature. The relative activity can be calculated as follows: relative activity (%) = (F_i_ − F_0_)/(F_t_ − F_0_) × 100%, where F_i_ and F_t_ refer to the fluorescence ratios in the presence and absence of 5-fluorouracil, respectively, and F_0_ refers to the fluorescence ratios in the absence of MTase.

## 3. Results

### 3.1. Principle of the MTase Detection

**Scheme 1:**
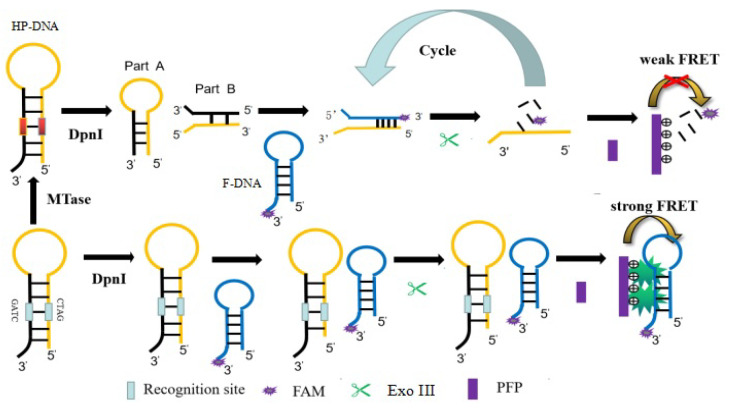
The principal of the method developed to detect MTase activity is based on exonuclease-III–aided FRET amplification.

The principle of the detection method is shown in Scheme 1. Two DNA strands are designed. One of them is a hairpin-shaped DNA (HP-DNA), which acts as a substrate for MTase. The stem region of the structure contains the MTase-specific recognition sequence GATC. The other strand is F-DNA labeled with the FAM fluorescent group at the 3ʹ end. Both DNA strands have >4 protruding bases at their 3ʹ end and thus cannot be cleaved by Exo III and can maintain a complete hairpin structure. In the presence of MTase and the endonuclease Dpn I, the GATC sequence in HP-DNA can be methylated by MTase, and the methylated HP-DNA can then be recognized and cleaved into two parts (parts A and B) by Dpn I. F-DNA can be hybridized with part A into dsDNA, which subsequently triggers the Exo III-mediated target recycling reaction [22]. After the Exo III cleavage process, numerous FAM molecules are released from F-DNA. The introduction of PFP leads to much weaker electrostatic interactions between the separated FAM and PFP. Thus, inefficient FRET from PFP to FAM was observed. In contrast, HP-DNA cannot be cleaved by Dpn I in the absence of MTase. Consequently, Exo III does not work, and when PFP are added, strong electrostatic interactions between PFP and F-DNA can induce efficient FRET from PFP to FAM labeled at the F-DNA probe. Therefore, based on the Exo III-aided signal amplification and FRET, MTase activity can be quantitatively detected through the change in the fluorescence signal. 

### 3.2. Feasibility of MTase Assay

**Figure 1:**
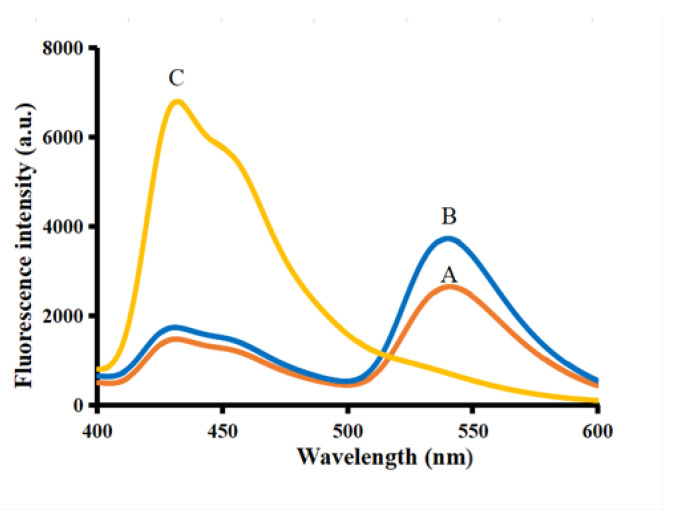
The feasibility of the developed method. (A) HP – DNA + DpnI + F – DNA + Exo + PFP; (B) HP – DNA + MTase + F – DNA + Exo III+PFP; (C) HP – DNA + MTase + DpnI + F – DNA + Exo III + PFP. Concentrations of HP-DNA, MTase, DpnI, F-DNA, Exo III and PFP were 50 nM, 40 U/mL, 60 U/mL, 100 U/mL, 5 U/mL and 0.8 μM. The reaction buffer (10 mM Tris-HCl, 50 mM NaCl, 10 mM MgCl_2_, and 1 mM DTT; pH 7.9) was used.

To assess the feasibility of a FRET-based strategy for the detection of MTase activity, three experimental conditions were designed. As shown in Figure 1, in the presence of MTase or Dpn I alone, the two HP-DNAs remained intact, and Exo III failed to cleave them. The FRET between the FAM and PFP occurred as normal, and thus the fluorescence intensities at 535 and 430 nm were enhanced and diminished, respectively (Curves A and B). When MTase and Dpn I were present, Dpn I could quickly cleave the methylated DNA, and the released part could hybridize with F-DNA to form a double strand that could be digested by exonuclease III, whereby the FAM fluorescent group could be released. Consequently, the FRET between the FAM and PFP was lost. The fluorescence intensity at 535 nm was weakened, and the fluorescence intensity at 430 nm was significantly enhanced (Curve C). These results show that a simple and effective MTase-detection assay based on exonuclease-III-aided signal amplification and FRET was established.

### 3.3. Optimization of Experimental Conditions

**Figure 2:**
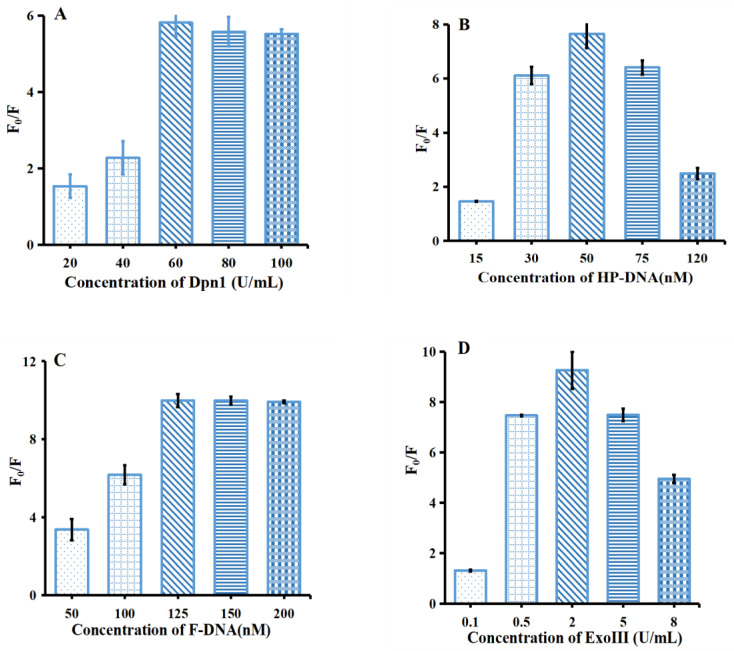
Optimization of experimental conditions. (**A**) concentrations of DpnI (20, 40, 60, 80, 100 U/mL); (**B**) concentrations of HP-DNA (15, 30, 50, 75, 120 nM); (**C**) concentration of F-DNA (50, 150, 125, 150, 200 nM); (**D**) concentrations of Exo III (0.1, 0.5, 2, 5, 8 U/mL). Error bars were calculated from three replicate measurements. F_0_ and F were the fluorescence ratio of the detection system without and with MTase, respectively.

Several experiments factors were optimized for the highest sensitivity. First, the concentration of Dpn I was optimized. As shown in Figure 2A, the fluorescence intensity reached a plateau when the concentration of Dpn I was 60 U/mL. Therefore, 60 U/mL Dpn I was used in the following experiments. We then optimized the concentrations of HP-DNA and F-DNA. The fluorescence intensity increased with increasing HP-DNA and F-DNA concentrations and reached the maximum at 50 nM HP-DNA and 125 nM F-DNA. Accordingly, these concentrations were used in the subsequent experiments. Finally, the concentration of Exo III was optimized. As can be seen in Figure 2D, the fluorescence intensity gradually increased with increasing concentrations of Exo III and reached the maximum at 2 U/mL Exo III. Thus, 2 U/mL of Exo III was used in the following experiments. 

### 3.4. Assay of MTase Activity

**Figure 3:**
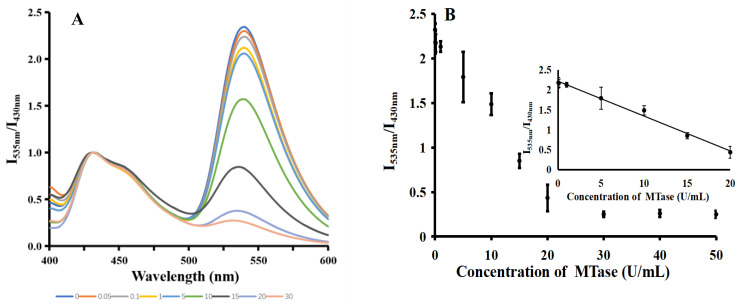
(**A**) Fluorescence emission spectra in the presence of increasing amounts of MTase (0, 0.05, 0.1, 1, 5, 10, 15, 20, 30 U/mL). (**B**) The relationship between fluorescence ratios and MTase activity. Error bars were estimated from three replicate measurements. The inset shows the linearity of the fluorescence ratios with respect to MTase concentrations (R^2^ = 0.9907).

In this part, various concentrations of MTase from 0 to 30 U/mL were added to the reaction system under the optimal experimental conditions. The ratio of fluorescence intensity was found to decrease as the MTase concentration increased (Figure 3). As the Dam MTase concentration increased, a growing number of methylated HP-DNA were cleaved by DpnI to form part A, which can trigger the digestion reaction of F-DNA by Exo III to inhibit the FRET from PFP to FAM. Then, more and more reporter probes can be cleaved, resulting in Dam MTase concentration-dependent fluorescence decrease. When the MTase concentration increased from 0.05 to 20 U/mL, the ratio of I_535__nm_/I_430 nm_ had a relatively good linear relationship with the MTase activity (R^2^ = 0.9907). The limit of detection (LOD) of the developed method was estimated at 0.045 U/mL according to the 3σ. This LOD value is even lower than that of electrochemiluminescence methods based on methylene blue [9], colorimetric methods based on G-quadruplex [23], fluorescence methods based on a novel methylation-responsive DNAzyme strategy [24], or a fluorescence method based on 2-aminopurine and thioflavin T [25,26], which was attributed to the improved amplification efficiency. Accordingly, the detection limit of the method developed in this study is comparable or even superior to that of most methods used for MTase detection in recent years (Table 1).

### 3.5. Specificity of the Method for Detecting MTase Activity

**Figure 4:**
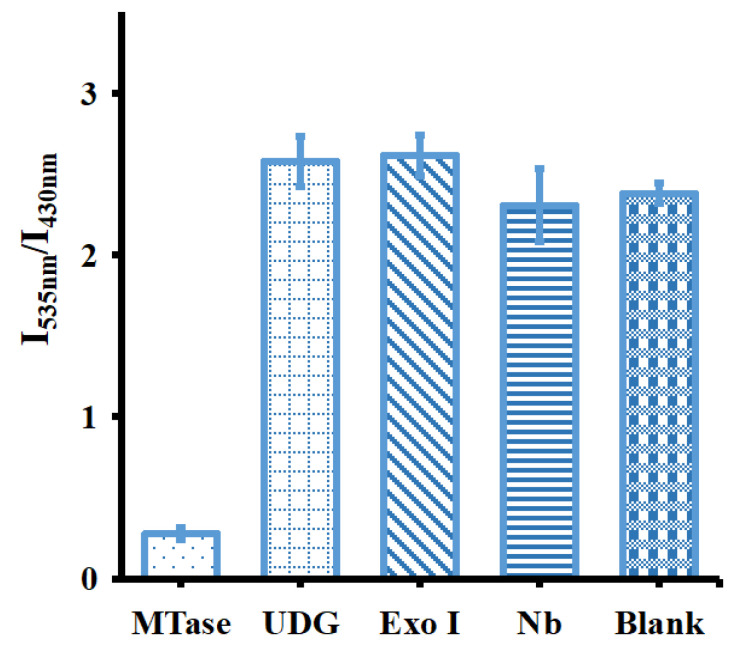
Fluorescence ratios of the biosensor in presence of UDG, Exo I, and Nb.BTSI. Error bars were estimated from three replicate measurements.

To evaluate the selectivity of the developed method, 40 U/mL UDG, Exo I, and Nb.BTSI were used as an interfering enzyme. As shown in Figure 4, only MTase could highly decrease the fluorescence ratio, whereas the other three enzymes had no obvious effect. Therefore, the method exhibited a good specificity for MTase against the selected interfering enzymes.

### 3.6. MTase-Activity Inhibition Assay

**Figure 5:**
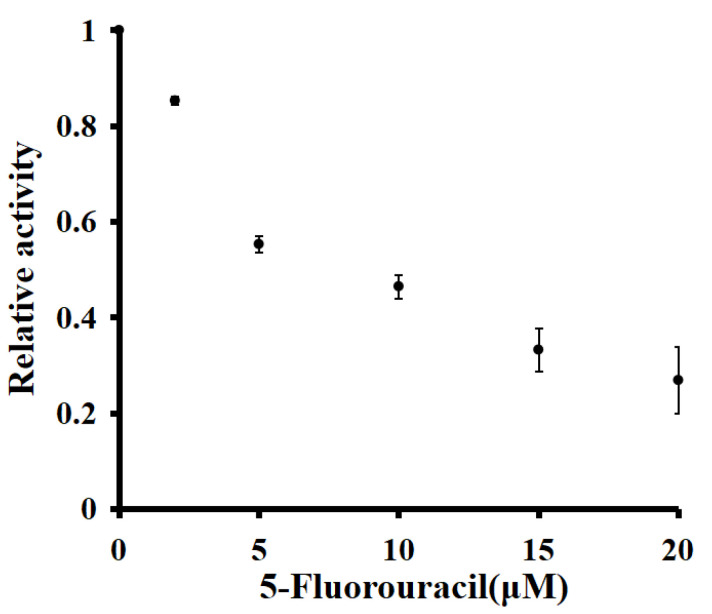
Effect of 5-fluorouracil on MTase activity. Error bars were estimated from three replicate measurements.

As an inhibitor of thymidylate synthetase, 5-fluorouracil was used to inhibit MTase. This nucleotide analog interferes with DNA synthesis by blocking the conversion of deoxyribouridine to thymidylate. According to the report by Ma and Tang et al., 5-fluorouracil has no significant effect on the activity of Dpn I and Exo III [27,28]. Then, we added various concentrations of 5-fluorouracil to the reaction. As shown in Figure 5, the relative activity of 40 U/mL MTase decreased as the concentration of 5-fluorouracil increased, and the halfmaximal inhibition concentrations (IC_50_) for 5-fluorouracil was estimated to be 10.02 μM. These results indicate that the method that we developed can be used for screening for MTase inhibitors, and that it has a good application prospect in the diagnosis and treatment of diseases.

### 3.7. Detection of the MTase Activity in Complex Biological Samples

To assess whether the developed method can be used for detecting the MTase activity in complex samples, MTase at various concentrations (including 5, 10, and 15 U/mL) was added into the 100-fold diluted spiked serum samples. As shown in Table 2, the MTase concentration was recovered in the range of 93.98–105.19%. These results indicate that the MTase-detection method we developed can be used on complex biological samples.

## 4. Conclusions

In conclusion, we developed an Exo-III–mediated target recycling strategy for the sensitive and specific detection of MTase activity. By taking advantage of the PFP strategy, the LOD of our strategy can be as low as 0.045 U/mL. In addition, since MTase recognizes a specific site, this enzyme can be well distinguished from any interfering enzyme. We successfully used the developed method to screen for MTase inhibitors. Furthermore, we demonstrated that the sensing platforms work under serum-poor conditions efficiently. Given the good analytical characteristics, the method developed in this study might have broad application prospects in biomedical research, disease diagnosis and treatment, and drug screening.

## Figures and Tables

**Table 1:** Comparison of reported methods for MTase detection.

Methods	Materials	LOD (U/mL)	Dynamic Range (U/mL)	Reference
Electrochemical	Au nanoparticle	0.02	0.075–30	[9]
Electrochemical	Methylene blue	0.07	0.1–10	[6]
Colorimetric	G-quadruplex	6	6–100	[23]
Fluorescence	Ag nanocluster	0.1	0.4–20	[24]
Fluorescence	2-Aminopurine	0.2	0.2–40	[25]
Fluorescence	Thioflavin T	0.1	0.1–8	[26]
Fluorescence	PFP	0.045	0.5–20	This work

**Table 2:** Recovery experimental results of human serum samples (*n* = 3).

Sample	Added (U/mL)	Found (U/mL)	Recovery (%)	RSD (%)
1	5	4.70 ± 0.24	93.98%	5.17
2	10	9.79 ± 0.30	97.91%	3.09
3	15	15.78 ± 0.56	105.19%	3.58

## Data Availability

Not applicable.
